# Convertible MRI contrast: Sensing the delivery and release of anti-glioma nano-drugs

**DOI:** 10.1038/srep09874

**Published:** 2015-05-12

**Authors:** Liang Zhang, Zhongwei Zhang, Ralph P. Mason, Jann N. Sarkaria, Dawen Zhao

**Affiliations:** 1 Radiology, UT Southwestern Medical Center, Dallas, TX; 2 Radiation Oncology, Mayo Clinic, Rochester, MN

## Abstract

There is considerable interest in developing nanohybrids of imaging contrast agents and drugs for image-guided drug delivery. We have developed a strategy of utilizing manganese (Mn) to enhance the nano-encapsulation of arsenic trioxide (ATO). Formation of arsenite (As^3+^)-Mn precipitates in liposomes generates magnetic susceptibility effects, reflected as dark contrast on T_2_-weighted MRI. Intriguingly, following cell uptake, the As-Mn complex decomposes in response to low pH in endosome-lysosome releasing ionic As^3+^, the active form of ATO, and Mn^2+^, the T_1_ contrast agent that gives a bright signal. Glioblastoma (GBM) is well known for its high resistance to chemotherapy, e.g., temozolomide (TMZ). Building upon the previously established phosphatidylserine (PS)-targeted nanoplatform that has excellent GBM-targeting specificity, we now demonstrate the effectiveness of the targeted nanoformulated ATO for treating TMZ-resistant GBM cells and the ability of the convertible Mn contrast as a surrogate revealing the delivery and release of ATO.

Glioblastoma multiform (GBM), the most lethal intracranial cancer, is characterized by densely populated tumor cells, high mitotic activity, profound angiogenesis and intratumoral necrosis^1^,^2^,^3^. The standard of care treatment for GBM is surgical resection followed by concurrent chemotherapy with temozolomide (TMZ) and radiation^2^,^4^. Despite the improvement in GBM survival when adding TMZ, recurrences are inevitable. A subset of tumor cells has recently been identified as the source of the recurring tumor cells after TMZ is administered to transiently arrest glioma growth, suggesting the existence of endogenous TMZ-resistant cells or cancer stem-like cells in GBM^5^,^6^. Several recent studies have shown that arsenic trioxide (ATO) is able to reverse GBM resistance by depleting the cancer stem-like cell population^6^,^7^,^8^. ATO is approved by the Federal Drug Administration (FDA) for the treatment of acute promyelocytic leukemia. ATO has also demonstrated significant activity in treating solid tumors. Via its effect on multiple cellular pathways, ATO induces apoptosis, inhibits cell proliferation and tumor angiogenesis, and promotes cell differentiation^9^. ATO in combination with TMZ and radiotherapy is currently being evaluated in clinical trials of glioma patients^10^. However, clinical efficacy of ATO on solid tumors has generally been limited by its systemic cytotoxicity.

Nanoparticles (e.g., liposomes, micelles, dendrimers) are emerging as promising drug-loading platforms with significantly increased drug payloads and additional benefits including prolonged circulation time and reduced adverse effects, as compared to free drugs^11^,^12^,^13^,^14^. Several FDA-approved drug preparations utilizing liposomes as the drug carriers, for instance, liposomal doxorubicin (Doxil), have shown promise in treatment of various cancer types in the clinic^15^. However, previous attempts to load ATO directly into liposomes suffer from low encapsulation efficiency and rapid leakage of ATO. Recently, O’Halloran and coworkers have shown a successful strategy of nano-encapsulated ATO by utilizing transition metals such as nickel or copper to actively load ATO into liposome. Nickel or copper and ATO form a complex in the core of liposome, preventing the leakage of ATO from liposomes^16^,^17^. The complex is stable at neutral pH, whereas it releases the active As^3+^ at low pH^17^.

One emerging nanotechnology with enormous potential for image-guided anticancer therapy involves the hybrid of imaging and therapeutic agents into a single nanostructure (so-called theranostics). Utilizing the transition metal approach, we have recently developed a novel nanohybrid of arsenite complex with manganese. We chose to use Mn to entrap ATO into liposome because Mn provides paramagnetic MRI contrast. With five unpaired electrons, Mn^2+^ is among the best T_1_ contrast agents^18^,^19^,^20^. However, the formation of As-Mn precipitates in the core of liposomes possesses magnetic susceptibility effects, resulting in a dark signal on T_2_-weighted MRI. Intriguingly, after the cell uptake and exposure to the low pH in endosome-lysosome system, the As-Mn complex decomposes to release ionic As^3^, the active form of ATO and Mn^2+^, which gives a bright signal on T_1_-weighted images. Thus, the convertible MRI contrast of Mn can serve as a surrogate of delivery and release of free ATO from its inactive nanoformulation (illustrated in Fig. 1).

We have previously established a phosphatidylserine (PS)-targeted nanoplatform for sensitive tumor imaging^21^,^22^. PS, the most abundant anionic phospholipid of the cell membrane, is normally constrained to the inner plasma membrane. Recent studies by us and others have shown that the harsh tumor microenvironment characterized by hypoxia, acidosis and oxidative stress causes redistribution of PS from the inner to the outer membrane leaflet of tumor endothelial cells and tumor cells in various cancers including GBM^23^,^24^,^25^. These PS-exposed tumor cells are actually found to be viable and not subjected to apoptotic process. PS exposure on tumor cells is inducible and reversible; the cells can resume growth and reestablish phospholipid asymmetry, which is distinct from the irreversible process occurring in cell death^26^,^27^. In normal mammalian cells, essentially all the PS localizes in the cell’s inner membrane. Thus, PS exposed on tumor, but not normal cells creates a highly specific tumor biomarker. By functionalizing the PEGylated liposomes with the F(ab’)_2_ fragments of PGN635, a novel human monoclonal PS-targeting antibody, our initial applications have clearly demonstrated that the PS-targeted nanoprobes of optical/MRI contrast agents provided sensitive and specific tumor imaging^21^,^22^. In the present study, we have utilized this nanoplatform for targeted delivery of our newly developed As-Mn nanohybrids to PS-exposed GBM cells (Fig. 1b). The GBM cells, which originated from clinically resected GBM that showed differential response to TMZ treatment, were tested with the treatment of PS-targeted nanoformulated ATO. Longitudinal MRI was applied to monitor the imaging contrast changes, aiming to achieve MRI-guided delivery and release of ATO.

## Results

### Preparation and characterization of PS-L-AsMn

**Figure 2:**
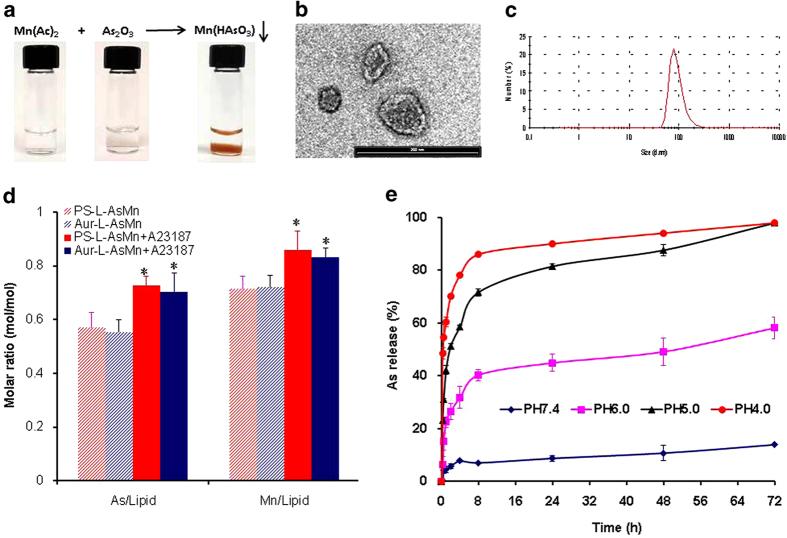
Characteristics of the PS-L-AsMn nanoparticle. (**a**) Brown precipitates were clearly seen after ATO was added to manganese acetate solution (**b**) A representative transmission electron microscopic (TEM) image (bar = 500nm) of PS-L-AsMn revealed the electron-dense precipitates in the cores (**c**) A size distribution curve obtained by dynamic light scattering (DLS) analysis indicates an average hydrodynamic size of PS-L-AsMn = 92.4 ± 2.6 nm (**d**) The liposome encapsulation ratios of As and Mn to lipid with/without ionophore A23187 were determined. The data indicated that significantly increased encapsulation efficiency was achieved when ionophore A23187 was applied (0.73 ± 0.03 and 0.85 ± 0.06 versus 0.57 ± 0.05 and 0.71 ± 0.04, respectively; * p < 0.05). Similar encapsulation ratios were obtained for the control antibody labeled liposomal AsMn (Aur-L-AsMn) (**e**) Extra-liposomal buffer was adjusted to various pHs ranging from 7.4 to 4. The pH-dependent and time-dependent release of As^3+^ from the liposome was determined.

As shown in Fig. 2a, manganese acetate and ATO form brown precipitates at room temperature. ATO is actively loaded into the core of liposomes, where the pre-entrapped manganese acetate complexes with ATO, as clearly seen in the TEM images as the electron-dense precipitates in the liposomal cores (Fig. 2b). Besides the passively loaded Mn^2+^, ionophore A23187, which is known as a mobile ion-carrier, was used to facilitate the loading of Mn^2+^across the liposome membrane and thus attract more ATO into the liposomes. A23187 has been shown to be most selective for Mn^2+^ among the divalent ions^28^. Indeed, this approach yielded the significantly enhanced encapsulation of both manganese and arsenite (0.85 and 0.73 molar to 1 molar lipid, respectively; Fig. 2d), as compared to that without A23187 (0.70 and 0.56; p < 0.05; Fig. 2d). As-Mn complex prevents the efflux of As at a neutral pH with only 8%, 10% and 13% of the total As leaking out at 24 h, 48 h and 72 h, respectively (Fig. 2e). However, the leakage started to increase significantly after lowering the extra-liposomal pH to 6, resulting in a 40%, 45% and 58% As loss at 24 h, 48 h and 72 h, respectively (Fig. 2e). The release rate increased with the decreased pH, for example, 82% at pH 5 and 90% at pH 4 after 24 h (Fig. 2e). Compared with the shuttling As ions, the transitional divalent metals (Mn^2+^) have much lower membrane permeability. As shown in Fig. S1, there was less than 5% leakage of Mn^2+^ after 72 h at various pHs. These data are in good agreement with previous studies of liposome containing nickel or copper-As by Chen and co-workers^17^. PS-L-AsMn was characterized with respect to particle size, charge, encapsulation efficiency and antibody coupling efficiency. The data are summarized in Table 1 and shown in Fig. 2c.

### Measurements of MRI relaxivity and convertible MRI contrast of manganese

**Figure 3:**
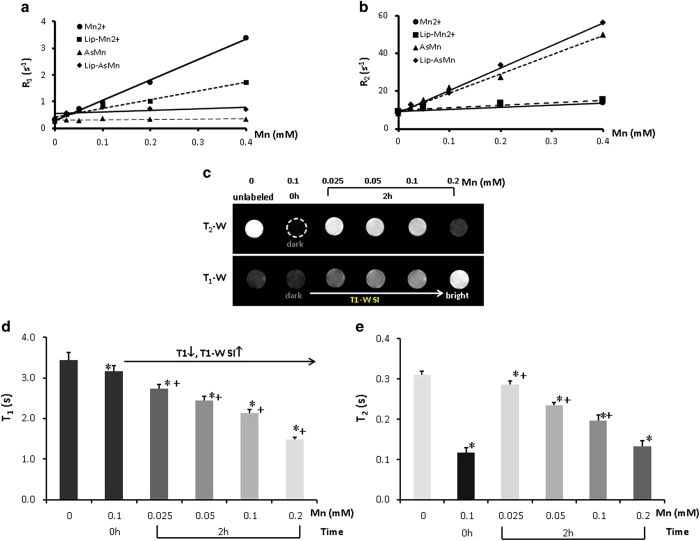
Conversion of dark to bright Mn contrast indicates the release of active As^3+^ (**a**) MRI relaxivity r1 of Mn^2+^, liposomal Mn^2+^ (Lip-Mn^2+^), As-Mn precipitates (AsMn) and liposomal AsMn (Lip-AsMn) was determined as 7.6, 3.3, 0.1 and 0.6 (mM^−1^s^−1^), respectively (**b**) The corresponding transverse relaxivity r2 was 11.3, 13.5, 97.9 and 108.7 (mM^−1^s^−1^) (**c**) GBM16 cells were cultured in the medium containing various concentrations of Lip-AsMn (ranging from 0 to 0.2 mM Mn). Two hours after incubation, 3 × 10^5^ cells of each concentration were suspended in their own medium (0.1 ml) and then mixed homogenously with 0.8% agarose in 96-well-dish. As a control, the identical number of GBM16 cells without the Lip-AsMn treatment were suspended in the medium containing 0.1 mM Lip-Mn^2+^ (0.1 ml) and mixed immediately with agarose. Subsequently, T_2_- and T_1_-weighted MR images were acquired. For the control cells (0 h, 0.1 mM), a signal void was seen on T_2_-w images (dotted circle). However, after incubation for 2 h, bright T_1_-w signals appeared and became greater with increased Lip-AsMn accompanied by significantly reduced susceptibility effect. (**d**) Quantitative T_1_ relaxation time measurements revealed a dose-dependent T_1_ shortening (**e**) Initially shortened T_2_ relaxation time due to the susceptibility effects recovered after the 2 h incubation, indicating the release of Mn^2+^. T_2_ decreased with the increased Mn^2+^ release. * p < 0.05, versus the unlabeled cells; + p < 0.05, versus the control (0 h, 0.1 mM).

Distinct MRI relaxivity between free Mn^2+^ ions and Lip-AsMn was observed at 9.4 T (Fig. 3a and b). The stable Lip-AsMn at a neutral pH and 37 °C possesses a high r2 (108.7 mM^−1^s^−1^) and a low r1 (0.6 mM^–1^s^–1^), as opposed to Mn^2+^ ions in Mn(Ac)_2_ that has a high r1 (7.6 mM^–1^s^–1^), a low r2 (11.3 mM^−1^s^−1^). The magnitude of the increase in r1 is greater than 12-fold, making it a highly sensitive convertible contrast agent, which is clearly reflected on switchable MRI signals. To test the switchable MRI contrast in response to a lower pH, the solution of the above 4 materials containing 0.1 mM Mn was adjusted to pH 5. Two hours later, the precipitates in AsMn were found to dissolve completely. MRI measurements clearly showed that T_1_ MRI contrast of both AsMn and Lip-AsMn switched from the initially dark to bright, while the initial bright contrast of Mn^2+^ and Lip-Mn^2+^ sustained (Fig. S2). To be noted, in common with the release assay data (Fig. 2e), the liposome encapsulated AsMn precipitates dissolved partially after the 2 h incubation, resulting in the MRI contrast change smaller than that of the naked AsMn (Fig. S2). Consistent with the T_1_ contrast changes, T_2_–w images showed that the initially dark contrast of AsMn and Lip-AsMn due to T_2_ susceptibility effect diminished (Fig. S2). Similar MRI contrast conversions were also observed in the following in vitro cell studies. As shown in Fig. 3c–e, a dark signal on T_2_-w was seen immediately after mixing Lip-AsMn and glioma cells due to the susceptibility effect of the AsMn precipitates. However, 2 h after the incubation, a bright signal appeared on T_1_-w MR images and became greater with increased concentrations of Mn, clearly indicating the non-specific cellular uptake of Lip-AsMn and subsequent release of T_1_ contrast Mn^2+^ ions after exposure to the low pH in endosome-lysosome system. Accompanying with the increased T_1_ contrast (Fig. 3c and d), T_2_ decreased with increased concentrations of Lip-AsMn (Fig. 3c and e), in particular, at the highest concentration of 0.2 mM, a dark T_2_-w signal was obvious (Fig. 3c). Concurrent with the results of Lip-AsMn in acidic solution (Fig. S2), the dynamic contrast changes resulted most likely from the combination effects of the remaining AsMn complex (incomplete dissolution) that possesses T_2_ susceptibility effect and the increased Mn^2+^ concentrations that also shorten T_2_. Indeed, prolonged incubation for 24 h led to more Mn^2+^ dissociated from the complex and thus even greater T_1_ contrast change (data not shown), as compared to the 2 h incubation time (Fig. 2c–e).

### PS-targeted delivery of ATO

**Figure 4:**
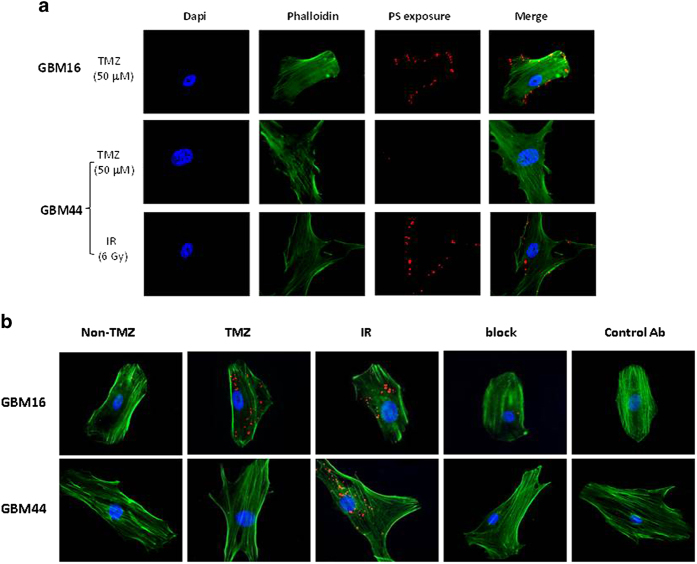
Specificity of PS-L-AsMn (**a**) TMZ-sensitive GBM16 cells were treated with 50 μM TMZ for 24 h before PGN635 antibodies were incubated with the cells for 1 h. Merge of the cytoskeleton-staining (green) and the PS-staining (red) indicated abundant PS exposure on the cell membrane. By contrast, TMZ did not induce PS exposure in TMZ-resistant GBM44 cells. However, a single dose of 6 Gy radiation caused marked PS exposure (**b**). Endocytosis after specific binding of rhodamine (Rho) embedded PS-L-AsMn was confirmed by visualizing cytoplasmic Rho (red) in GBM16 with TMZ or IR and GBM44 only with IR. Rho signal was not seen in untreated cells (Non-TMZ) or with the control Ab labeled liposome. Pretreatment with PGN635 blocked the PS-binding of PS-LAsMn.

Building on the PS-targeted nanoplatform previously established in our lab^21^,^22^, we have developed the PS-L-AsMn by functionalizing the surface of PEG-liposomes with PGN635 F(ab’)_2_, aiming to achieve the targeted delivery of ATO to GBM cells. As shown in Fig. 4a, in response to TMZ, the TMZ-sensitive GBM16 cells presented massive PS exposure on their cell membrane, detectable using PS-targeting antibody, PGN635, while minimal PS exposure was found on the TMZ-resistant GBM44 cells. However, a single dose of 6 Gy irradiation caused marked PS exposure on GBM44 cells. To test its targeting specificity, rhodamine labeled PS-L-AsMn was incubated with those PS exposed GBM cells for 1 h. Intracellular rhodamine signals were clearly seen in GBM16 cells treated with TMZ or IR and GBM44 cells treated with IR only. There was essentially no rhodamine signal observed from the control cells without treatment-induced PS exposure or GBM44 cells treated with TMZ (Fig. 4b). The binding specificity of PS-L-AsMn was further confirmed by the results of the control Ab labeled Aur-L-AsMn or pretreatment with PGN635 to block the binding of PS-L-AsMn (Fig. 4b).

**Figure 5:**
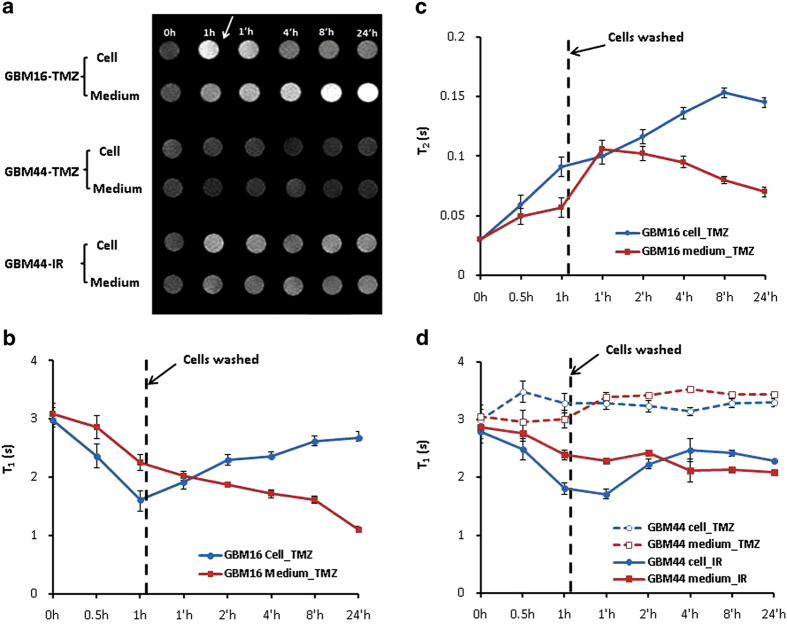
Dynamic MRI monitoring of specific targeting of PGN-L-AsMn to PS exposed GBM cells (**a**). TMZ-sensitive GBM16 cells were treated with TMZ for 24 h before incubation with PS-L-MnAs (10 μM Mn) for 1 h and then washed to remove the unbound PGN-L-AsMn (arrow). The cells were continued to culture in the fresh medium without PGN-L-AsMn for up to 24 h (‘h). T_1_-weighted images were acquired separately for the cells and their culture medium at different time points. The dark signals of both the cells and their medium at 0 h became brighter at 1 h. After extracellular unbound PS-L-MnAs was washed away at 1 h, the cellular signal decreased with time. However, the extracellular signal increased with time, implicating increased trafficking of Mn^2 + ^from the cells to extracellular medium. There was no obvious signal change in the TMZ-treated GBM44 cells. However, radiation-treated GBM44 cells showed the signal change pattern similar to that of TMZ-treated GBM16 cells. (**b**) T_1_ relaxation time was also obtained for the above studies, which coincided with signal changes in **a. (c)**. Conversion of the As-Mn precipitates to Mn^2+^ ions was further supported by the increase on T_2_ relaxation time (**d**) There was no significant change in T_1_ of the GBM44 cells treated with TMZ. However, radiation induced the PS exposure of the GBM44 cells, resulting in T_1_ shortening.

**Figure 6:**
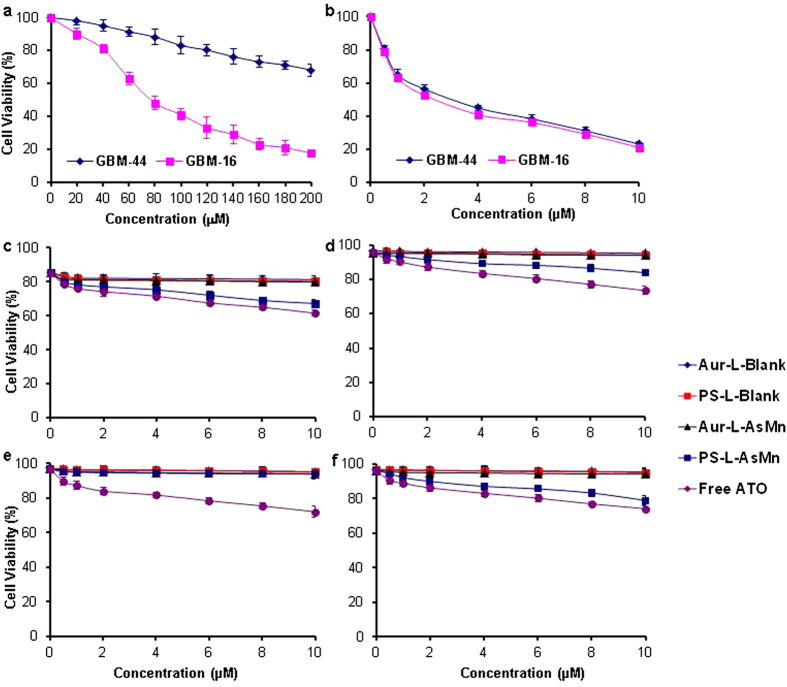
*In vitro* cytotoxicity of ATO in TMZ-sensitive or resistant glioma cells. GBM-16 and GBM-44 glioma cells showed differential response to TMZ (**a**), but responded equally well to free ATO (**b**). **c-f.** GBM16 (**c** and **d**) and GBM44 **(e** and **f**) cells were pretreated with TMZ (50 μM, **c** and **e**) or irradiation (6 Gy, **d** and **f**) 24 h before incubation with free ATO or various liposomal formulations for 1 h. The cells were then washed and continued to culture in new medium. The cell survival assay at 72 h revealed that the free ATO had the highest cell killing in each group and acted in a dose-dependent manner. Compared to the non-specific Aur-L-AsMn, PS-L-AsMn resulted in higher cell death in the GBM16 cells pretreated with TMZ (**c**) or IR (**d**) and the GBM44 cells with IR (**f**), but not in the TMZ treated GBM44 (**e**).

We have further applied MRI to determine if the observed T_1_ and T_2_ changes will be sensitive enough to assess specific binding, cellular uptake and release kinetics. As clearly seen in Fig. 5a, after 1 h incubation with GBM16 cells pretreated with TMZ, the original dark signals of the cells became brighter, as compared to a modest increase in signal intensity of the culture medium, indicating that intracellular Mn^2+^ trafficking predominated during this period. Quantitative T_1_ measurements revealed a 50% decrease in T_1_ of the cells at 1 h (Fig. 5b). Accompanied by increased Mn^2+^, the susceptibility effect decreased, which was seen as increased T_2_ (Fig. 5c). These MRI data indicated that the dissociation of the As-Mn complex to release Mn^2+^ and As^3+^ started to occur shortly after the incubation. After removing the unbound PS-L-AsMn from the medium at 1 h, MRI measurements were continued, to evaluate the release kinetics. Intriguingly, T_1_-w images revealed that signal intensity decreased gradually in the cells, but increased in the medium during the longitudinal observation up to 24 h (Fig. 5a). Quantitative T_1_ measurements were in good accordance with the SI changes (Fig. 5b). Similar dynamic changes in MRI contrast were also observed in the GBM44 cells pre-treated with irradiation but not TMZ, further confirming the targeting specificity of PS-L-AsMn (Fig. 5d). Despite some cell death that may contribute to the MRI contrast changes, it is unlikely that significant numbers of the cells died at earlier time points after a short period of 1 h incubation with PS-L-AsMn. Our cell viability studies revealed that significant difference in cell survival occurred 72 h after treatment (Fig. 6). Thus, the observed MRI contrast changes resulted primarily from the intracellular conversion of the As-Mn complexes to Mn^2+^ and the trafficking of Mn^2+^ from intracellular to extracellular space. Atomic absorption spectrometry confirmed that the contents of both Mn and As in the medium increased with time (data not shown), indicating intra- and extra-cellular trafficking of both ions. T_2_ data of GBM44 cells pre-treated with TMZ or IR showed that sharper increase in T_2_ of TMZ-treated cells than that of IR-treated cells after removing the unbound PS-L-AsMn, indicating that the TMZ-treated GBM44 cells had minimal uptake of PS-L-AsMn (Fig. S3). The results were consistent with the observations that GBM44 cells resisted to TMZ but responded to IR. All the MRI results further supported the specific targeting of PS-L-AsMn.

### Cytotoxicity of PS-L-AsMn

In agreement with previous Mayo clinic studies^29^, GBM16 and GBM44 cells showed differential response to TMZ treatment with an IC_50_ = 78 μM and >300 μM, respectively (Fig. 6a). However, both of the cells responded equally well to ATO treatment with a similar IC_50_ = 2.4 and 2.8 μM, respectively (Fig. 6b). To evaluate the cytotoxic effect of PS-L-AsMn, GBM16 and GBM44 cells were pretreated with TMZ or IR to induce PS exposure before the cells were incubated with free ATO or ATO encapsulated PS-L-AsMn or Aur-L-AsMn. To study the cytotoxicity due to the specific targeting of PS-L-AsMn, the cells were washed after a short period of 1 h incubation to remove the remaining unbound drugs from the medium and the cell survival was evaluated at 72 h. As expected, the cells treated with free ATO in each group had the highest cell death (Fig. 6c–f). For the cells pretreated with TMZ, PS-L-AsMn treatment resulted in higher percentages of GBM16 cell death than those of Aur-L-AsMn, and significant difference was observed at the concentration of 6 μM (28%) and above (31% at 8 μM and 33% at 10 μM), as compared to the corresponding Aur-L-AsMn groups (p < 0.05; Fig. 6c). The cell death (~20%) observed in each of the control groups was attributed to the TMZ pretreatment. By contrast, for the GBM44 cells pretreated with TMZ, without the significantly predisposed PS exposure, there was no significantly higher cell death induced by PS-L-AsMn (Fig. 6e). For the IR-treated cells, however, both cell lines responded to PS-L-AsMn with higher cell death, compared to the Aur-L-AsMn groups (Fig. 6d and f). These data were consistent with the immunohistochemical findings previously shown in Fig. 4b, supporting that PS-L-AsMn enables PS-targeted delivery and intracellular release of active ATO to exert its cytotoxic effect. Prolonged incubation time (4 h) was also applied in a subset of the GBM cells. Despite the increased cell death observed for the PS-L-AsMn treated cells, the Aur-L-AsMn treatment also induced significantly higher cell death than that in the control groups (p < 0.05; Fig. S4), indicating that the non-specific cell uptakes of liposomal drugs occurred during the longer incubation time.

## Discussion

The efficacy of most systemic chemotherapeutics is limited by tissue toxicity and by physiologic barriers that prevent the delivery of therapeutic doses to the tumor. Polymer-based and liposomal drug delivery systems have been designed to increase tumor drug levels while limiting systemic drug exposure^15^. Various kinds of chemotherapeutic agents have been successfully encapsulated into these nanoparticles using either an equilibration method or remote loading methods. The approach of transition metal complexation has been previously introduced by Cullis and Balley^30^,^31^. A number of traditional chemotherapeutic agents including doxorubicin, topotecan and platinum have been successfully entrapped in liposomes by forming drug-metal complex^32^,^33^.

We chose to use manganese, a traditional MRI T_1_ contrast agent, to entrap ATO in the core of liposomes. It is well known that liposomes can be taken up by cells *via* clathrin-mediated endocytosis and then subjected to acidification along the endosomal pathway from early endosomes to lysosomes, which leads to destabilization of the liposome bilayer and release of encapsulated materials^11^. As clearly shown in Fig. 3, non-specific uptake by glioma cells resulted in decomposition of the As-Mn complexes, which was reflected as the convertible MRI contrast change from dark to bright. Similarly, Bennewitz *et al.* have recently reported that polymer PLGA encapsulated insoluble MnO crystals possesses a high T_2_* effect. However, T_2_* effect decreases, while T_1_ effect becomes dominant after cellular uptake^34^. In common with previous studies by others, liposome encapsulated Mn^2+^ has r1 = 3.3 mM^–1^s^–1^, which is >50% decrease in r_1_ as compared to the free Mn^2+^. The decrease is mainly due to limited exchange between intra-liposomal Mn^2+^ and outside water^35^. Taken together, the As-Mn precipitates encapsulated in liposome at a neutral pH acts predominantly as negative T_2_* or T_2_ contrast, while Mn^2+^ released from the liposomes at an acidic pH becomes positive contrast, eliciting bright contrast on T_1_-w images.

All the MRI investigations in this study were conducted on the high field, 9.4 T magnet. It is known that the same MRI contrast agents vary in relaxivity and thus relaxation time at high MR field strength (9.4 T) versus the clinical field strength (1.5 T or 3 T), *e.g.* T_1_ is shorter at 1.5 T than at 3 T. For the typical T_1_ contrast agents, i.e., Gd-chelators or Manganese, the lower field strength is expected to work better with even higher contrast enhancement achievable with the same administered doses and shorter repetition time^36^.

It is well recognized that the effectiveness of nano-drug is often governed by the kinetics of drug release after systemic administration^37^. Integration of imaging agents into nano-drug delivery systems aims to achieve the image-guided drug delivery^32^,^38^,^39^,^40^,^41^. Despite the considerable progress on developing such theranostic agents, achieving highly specific and sensitive tracking of the delivery and release of the nano-drugs *in vivo* and in real-time remains a formidable challenge. Typically for the dual functional nanoformulation, imaging probes and drugs are loaded separately into a different compartment of a nanocarrier, i.e., core and shell. Inconsistent release or leakage of the imaging agents or the drugs is often encountered after administration, hindering the imaging-based accurate monitoring of drug distribution. Dewhirst’s lab has recently utilized the strong acid salt, MnSO_4_ as the transition metal transporter to successfully load doxorubicin into the core of liposomes^32^,^42^,^43^. MRI was used to image T_1_ change induced by Mn^2+^. T_1_ data were converted to local Mn^2+^ levels and further correlated with the doxorubicin concentrations in tumor tissues. The correlation was based on the assumption that T_*2*_ effects can be neglected. However, it has been noticed in their studies that T_*2*_effects may influence the imaging-based measurements^42^. Given the dynamic process of Lip-AsMn dissociation, the approach of utilizing both T_1_ and T_2_ changes, as proposed in this study, should be superior to the one based on the sole T_1_ contrast in predicting both the delivery and release of As^3+^, the active form of ATO. Moreover, because both ions are cationic and have similar molecular weight, spatial localization of Mn^2+^ visualized by MRI may correlate better with biodistribution of As^3+^ than that of larger molecules such as doxorubicin or taxols.

Currently, delivering therapeutic agents to brain tumors is a major challenge. The stealth liposomal drugs have been shown to passively accumulate in tumor tissues via the leaky vasculature of solid tumors. Despite extensive angiogenesis in GBM, disruption of blood brain barrier (BBB) in GBM is heterogeneous, indicating many intratumoral regions still contain the intact BBB^44^,^45^. Nanocarriers with appropriate surface characterization are a promising approach for the delivery of therapeutics via BBB^14^,^46^. Taking advantage of several receptors expressed at the BBB, several ligands or antibodies that bind to such receptors have been utilized to conjugate directly or indirectly with therapeutic compounds, enabling receptor-mediated transport of drugs.

Clearly, discovery of a glioma-specific biomarker will be critical for development of glioma-targeted therapy. Moreover, this biomarker needs to be accessible to its ligands or antibody. Thus, a vascular luminal surface-exposed biomarker may be ideal. Our previous data have shown that the lipid, phosphatidylserine (PS), becomes exposed on the outer surface of vascular endothelial cells (ECs) of glioma due to oxidative stresses present in the tumor microenvironment^22^,^24^,^47^. In normal cells, even in highly angiogenic ovarian blood vessels, PS is asymmetrically distributed across the plasma membrane with essentially all the PS localized in the cell’s inner membrane. Therefore, PS-targeted liposomal nanocarrier has a potential to deliver therapeutic doses of ATO into GBM via both the non-specific EPR effect through the disrupted tumor vessels and the specific targeting of PS-exposed ECs of the intact tumor vessels and subsequent the cell-mediated transcytosis. Moreover, our previous studies have also found the significantly reduced uptake of PS-targeted liposomes by liver and spleen of the RES system^21^,^22^. Thus, significantly reduced systemic toxicity of ATO is anticipated with the PS-targeted liposomal delivery system.

The liposome formulations used are biologically inert, biocompatible, and cause little or no adverse toxic or antigenic reactions. Encapsulation of ATO in liposomes aims to reduce its significant systemic cytotoxicity. In addition to ATO, the toxicity of Mn remains an issue. The LD_50_ of a single dose of Mn^2+^ in rodents has been reported as 272 μmol/kg^48^. The dose used in the above *in vitro* studies (10 μM Mn) was well below the toxic dose, but proved to be sensitive enough to provide a strong imaging contrast. It has been documented in several studies that mice tolerated well 8 mg/kg i.p. ATO^6^,^49^,^50^. If this dose is used as the maximum dose for the future treatment, the corresponding Mn concentration of PS-L-AsMn will be 110 μmol/kg, which is also well below the LD_50_ dose. Given the tumor-targeted nanodelivery, the drug distribution in normal tissues is expected to be much lower, hereby, less toxic.

The standard of care of treatment for GBM is surgical resection followed by concurrent chemotherapy with TMZ and IR^2^,^4^. In addition to their own cytotoxicity, our data have clearly shown that TMZ or IR induced significant exposure of PS in the GBM cells including the TMZ-resistant GBM cells. Therefore, the combination treatment may overcome the single drug resistant tumors. Moreover, the unique acidic microenvironment in tumor will also facilitate the release of ATO at extracellular space of tumor cells. Taken together, it is highly expected that the combination of TMZ or IR with nanoencapsulated ATO will achieve synergistic effects on treating GBM.

In summary, we have developed a novel nanohybrid of cytotoxic ATO complexed with MRI contrast manganese, PS-L-AsMn, enabling the use of convertible MRI signals (dark to bright) to predict not only the delivery but also the release of ATO. The PS-L-AsMn is stable at a neutral pH, possessing MRI susceptibility effects (dark signal), while it releases Mn^2+^ (bright signal) and As^3+^ ions at an acidic pH. As^3+^, the active form of ATO, showed excellent cytotoxicity on both the temozolomide-sensitive and resistant GBM cells. Because PS exposure represents a specific biomarker of gliomas, as demonstrated in our previous studies, functionalizing the PEG-liposomes with PS-targeting antibodies allows the specific binding of PS-L-AsMn to PS-exposed glioma cells and subsequent release of active ATO in these cells. Our present study has built a foundation for *in vivo* application of PS-L-AsMn as a potential multi-functional imaging and therapeutic agent for glioma.

## Methods

### Preparation and characterization of liposomal encapsulation of arsenic trioxide

Egg phosphatidylcholine (EPC) (Sigma-Aldrich Corporation, MO), cholesterol (Sigma-Aldrich Corporation, MO), 1,2-distearoyl-sn-glycero-3-phosphoethanolamine-N-[methoxy(polyethylene glycol)_2000_] (PEG_2000_–DSPE) and 1,2-distearoyl-sn-glycero-3-phosphoethanolamine-N-[carboxy(polyethylene glycol)_2000_] (COOH-PEG_2000_-DSPE ) (Avanti Polar Lipids, Alabaster, AL) were dissolved in chloroform (55:48:5:1 μmol/μmol) in a pear-shaped flask. The lipid film was prepared by removing the chloroform using a rotary vacuum evaporator. The dry lipid films were then hydrated in 300 mM aqueous Mn (CH_3_COO)_2_ (Sigma-Aldrich) solution (3 mL) with sonication in water bath for 5 min, followed by sonication using a probe-type sonicator (Omni International Inc, Kennesaw, GA) for 5 min. The liposomes were then extruded through polycarbonate membranes (200 nm and 100 nm pore sizes, respectively, Nalgene, Rochester, NY, USA). To further enhance the [Mn (CH_3_COO)_2_] in liposomes, the ionophore A23187 (0.15 μg/μmol lipid; Sigma-Aldrich) and Mn (CH_3_COO)_2_ (900 mM) were added to the liposome solution and incubated at 50 °C for 30 min. The excess Mn (CH_3_COO)_2_ outside the liposomes was removed by quick spin Sephadex G-50 column (Fisher Scientific, Pittsburg, PA). Arsenic trioxide (ATO; 300 mM; Sigma-Aldrich) dissolved in sodium hydroxide (pH = 7.4) was added to the collected liposome solution and incubated at 50 °C for 60 min. The excess ATO in the solution was then removed by quick spin Sephadex G-50 column with PBS (pH 7.4). The loading efficiency of the Mn and As was determined by the Atomic absorption spectrometer (Varian Medical Systems, USA).

To functionalize the liposomes with PS-targeting antibody, the human monoclonal antibody PGN635 that was generated by Affitech A.S. (Oslo, Norway) in collaboration with Peregrine Pharmaceuticals. Inc., (Tustin, CA) was used. A human monoclonal antibody that binds to an irrelevant antigen (*S. Aureus* clumping factor A) was used as a negative control antibody. PGN635 and the control (Aur) Ab F(ab’)_2_ fragments were generated by reacting antibodies with pepsin at a molar ratio of 1:130 (antibody:pepsin) for 1 h at 37 °C. F(ab’)_2_ fragments (MW = 110 kD) were purified by FPLC using an S*-*200 column (Pharmacia, Piscataway, NJ) and PBS running buffer. PGN635 F(ab’)_2_ or Aur F(ab’)_2_ were then conjugated to the distant terminus of polyethylene glycol (PEG) chain coated liposomes, as described in details previously^21^,^22^. Briefly, 3.52 μmol of 1-(3-dimethylaminopropyl)-3-ethylcarbodiimide hydrochloride (EDC, Sigma-Aldrich) and 7.88 μmol of N-hydroxysuccinimide (NHS, Sigma-Aldrich) were added into 2 ml of the arsenic trioxide liposomes (COOH-PEG_2000_-DSPE:EDCI:NHS = 0.02:3.52:7.88,  μmol/ μmol) and gently stirred for 30 min at room temperature. Excess EDC and NHS were removed by dialysis. PGN635 F(ab’)_2_ or Aur F(ab’)_2_ (0.01 μmol) were then added into the suspensions and allowed to continuously react for 5 h at room temperature. Excess EDC and NHS were removed by dialysis. The liposome suspensions were applied to a quick spin Sephadex G-50 column equilibrated with PBS and centrifuged at 150 g to remove the uncoupled antibodies. Subsequently, liposome fractions were collected from quick spin Sephadex G-50 column, and the content of antibodies on the liposomes was determined by the bicinchoninic acid (BCA) kit (Sigma-Aldrich). To track the liposomes-cell interactions, 1,2-dioleoyl-sn-glycero-3-phosphoethanolamine-N-(lissamine rhodamine B sulfonyl)(ammonium salt) (Rh-PE) (Avanti Polar Lipids, Alabaster, AL) (0.5 mol %) was dissolved in chloroform and added to the liposomal formulations. The PS-targeted liposomal nanohybrids of As-Mn are referred as PS-L-AsMn or the control, Aur-L-AsMn throughout the paper.

The mean diameter and zeta potential of the liposomes were measured by dynamic light scattering (DLS) analysis with Zetasizer 3000HSA (Malvern Instruments Ltd., U.K). The morphological shapes were observed using a transmission electron microscope (TEM, JEM-1230, JEOL, Japan).

### pH-dependent release rate

PS-L-AsMn solutions (5 ml) were added into a semipermeable dialysis tube and were kept at 37 °C at different pHs. An extra liposomal buffer of PBS (100 ml) was used for maintaining the pH at 7.4. For pH 6.0, 5.0 and 4.0, PBS was adjusted accordingly with additional acetic acid. A volume of 0.1 ml PS-L-AsMn solutions was taken at various time points up to 72 h. Then, 0.05 ml samples were applied to a quick spin Sephadex G-50 column to remove As and Mn which had leaked from liposomes. The amount of As and Mn remained in liposomes was determined by the atomic absorption spectrometer. The As or Mn release percentage (%) was calculated as [(r_o_−r_i_)/r_o_] × 100%, where r_o_ is the initial amount of As or Mn and r_i_ is the amount of As or Mn in liposomes at a specific time point.

### Cell culture

Temozolomide (TMZ)-sensitive (GBM16) or resistant (GBM44) glioblastoma cells (Mayo Clinic) were cultured in Dulbecco’s modified Eagle’s medium (DMEM) with 1% penicillin/streptomycin (Sigma-Aldrich) and a low fetal bovine serum (2.5%; Invitrogen) to inhibit murine fibroblast growth. After the 4-week selection, the serum concentration was increased to 10%, and the GBM cells were then used for the following *in vitro* studies.

### Immunohistochemical analysis

#### Detection of exposed PS on the surface of GBM cells

To induce PS exposure, GBM16 and GBM44 cells were treated with either a single dose of 6 Gy X-radiation (IR) or TMZ (50 μM). Untreated cells were used as a control. Twenty-four hours later, the cells were fixed with 4% paraformaldehyde for 20 mins and incubated with full length PGN635 (1.5 μg/ml) for 1 h. PGN635 was detected using goat anti-human IgG conjugated to Cy3 (1:800) for 30 min. Cell membranes were permeabilized with 0.1% Triton X-100 (5 mins) and cytoskeletons were stained with Alexa Fluor 488–labeled phalloidin. Cell nuclei were counterstained with DAPI.

#### PS-specific targeting and internalization of PS-L-AsMn

A subset of the above pretreated GBM16 and GBM44 cells was incubated with PS-L-AsMn-Rhod or the control Aur-L-AsMn-Rhod at a concentration of 10 μM Mn for 1 h. For the blocking study, the cells were pretreated with the full length PGN635 for 1 h prior to PS-L-AsMn-Rhod. Unbound particles were washed away with PBS. The cells were then counterstained with phalloidin and DAPI. Fluorescence microscopic observations at 40 X were undertaken.

### MRI measurements

#### MRI relaxivity

The samples (0.1 ml) of manganese acetate, liposome encapsulated manganese acetate (Lip-Mn^2+^), As-Mn precipitates or Lip-AsMn containing various concentrations of Mn were mixed homogeneously with 0.8% agarose in 96-well-dish. To measure the insoluble As-Mn complex, the complex was suspended in deionized water and sonicated by a probe-type sonicator to achieve the particle size with a mean diameter of 100 nm. The phantoms were imaged on a 9.4 T horizontal bore magnet with a Varian INOVA Unity system (Agilent, Palo Alto, CA). An inversion recovery pulse sequence (TR/TE = 4000/40 ms, Echo Train Length (ETL) = 8, Echo spacing = 10 ms, FOV = 50 mm × 50 mm with 128 × 128 acquisition matrix, inversion time (TI) = 10, 437, 864, 1291, 1718, 2145, 2572 and 3000 ms) was applied to measure T_1_ values. Fast spin echo multi-slice (FSEMS; TR = 3000 ms, effective TE ranging from 40 to 120 ms with a 20 ms increment, average = 2, number of slices = 2, acquisition time = 10 min) was used to measure T_2_ values. A linear correlation between relaxation rate of R_1_ or R_2_ and Mn concentrations was obtained for calculation of r1 or r2 relaxivity of each sample.

#### Convertible MRI contrast in acidic solution

The samples (0.1 mM Mn) of Mn^2+^, Lip-Mn^2+^, AsMn precipitates or Lip-AsMn were kept at acidic pH 5 and then mixed homogeneously with 0.8% agarose in 96-well-dish at 0 and 2 h. The above MRI procedures were performed to obtain T_1_- and T_2_-w images and quantitative T_1_ and T_2_ values for each mixture.

#### Convertible MRI contrast due to the cell uptake and release of Mn*2+*

GBM16 cells were cultured in the medium containing different concentrations of Lip-AsMn (ranging from 0 to 0.2 mM Mn) for 2 h or prolonged for 24 h. At 2 h or 24 h, the cells and their culture medium were collected separately. 3 × 10^5^ cells of each concentration were suspended in their own medium (0.1 ml) and then mixed homogenously with 0.8% agarose in 96-well-dish. As a control, the identical number of GBM16 cells without the Lip-AsMn treatment were suspended in 0.1 mM Lip-Mn^2+^ solution (0.1 ml) and mixed immediately with agarose. The above MRI procedures were performed to obtain T_1_- and T_2_-w images and quantitative T_1_ and T_2_ values for each mixture.

#### MRI of PS-targeted delivery and release of PS-L-AsMn

To study the PS-targeting specificity of PS-L-AsMn, the GBM16 and GBM44 cells that were pretreated with TMZ (50 μM) or IR (6 Gy) to induce PS exposure were incubated with PS-L-AsMn (10 μM Mn), as described above. Before and at 0.5 h and 1 h after incubation, the cells and their culture medium were collected separately. The agarose gels containing the cells (3 × 10^5^) or medium alone (0.1 ml) were made and then subjected to quantitative T_1_ and T_2_ measurements. To further study the PS-targeted delivery and release of PS-L-AsMn, a subset of the cells was incubated with PS-L-AsMn for 1 h. The cells were then washed twice with PBS to remove the unbound PS-L-AsMn, and continued to culture in the fresh medium without PS-L-AsMn for up to 24 h. At different times of 1, 2, 4, 8 and 24 h (‘h), the above mentioned procedure was applied to make the MRI agarose gels containing either the cells or their culture medium for T_1_ and T_2_ measurements.

### *In vitro* cytotoxicity

#### Cytotoxicity of ATO

The GBM16 and GBM44 cells were seeded into 96-well culture plates at a density of 5.0 × 10^3^ cells per well and grown in serum-containing culture medium in the incubator at 37 °C and in the presence of 5% CO_2_ for 24 h. TMZ (0–200 μM; purchased from Pharmacy) or free ATO (0–10 μM; Sigma-Aldrich) were added into the culture medium. The cells were cultured for 72 h and the cell survival was evaluated by the sulforhodamine B (SRB) staining assay, and the absorbance was read on a microplate reader at 540 nm. The survival percentages were calculated using the following formula: Survival% = (A_540nm_ for the treated cells/A_540nm_ for the control cells) × 100%, where A_540_ nm is the absorbance value. Each assay was repeated in triplicate. Finally, the dose-effect curves were plotted.

#### Cytotoxicity of PS-targeted PS-L-AsMn

To examine the cytotoxic effect of PS-L-AsMn, GBM16 and GBM44 cells were treated with TMZ (50 μM) or IR (6 Gy) in 96-well culture plates. Twenty-four hours later, the cells were washed and added with free ATO (0–10 μM), PS-L-ASMN (0–10 μM As), Aur-L-AsMn (0–10 μM As), PS-L-Blank or Aur-L-Blank (at equivalent lipid concentrations). At 1 h or 4 h, the cells were washed and then incubated in drug-free medium. The survival rate was measured at 72 h by the sulforhodamine B (SRB) staining assay, as described above.

### Statistical analysis

Analysis of quantitative data of MRI relaxivity and MRI relaxation time was performed on a home written MATLAB program on both pixel-by-pixel and region of interest (ROI) basis. Statistical methods including paired Student’s t-test and ANOVA were used to determine significance among groups.

## Author Contributions

L.Z. and D.Z. designed research; L.Z., Z.Z. and D.Z. performed the research; L.Z., Z.Z. and D.Z. analyzed data; L.Z., R.M., J.S., and D.Z. wrote the paper.

## Additional Information

**How to cite this article**: Zhang, L. *et al*. Convertible MRI contrast: Sensing the delivery and release of anti-glioma nano-drugs. *Sci. Rep.* **5**, 9874; doi: 10.1038/srep09874 (2015).

## Supplementary Material

Supplementary Information

## Figures and Tables

**Figure 1:**
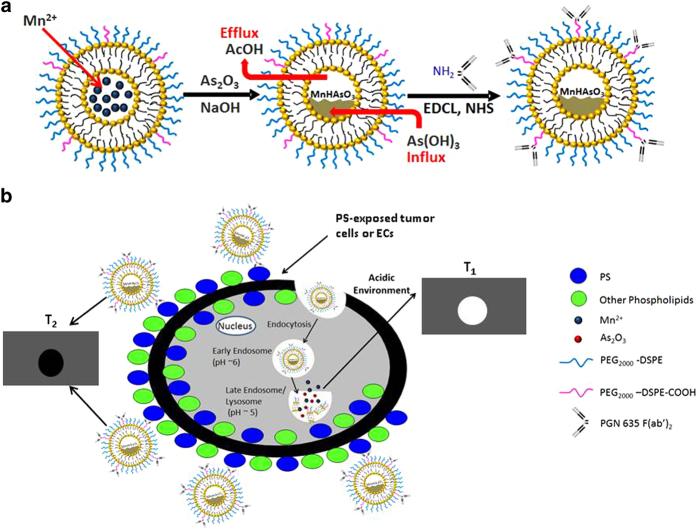
Convertible MRI contrast of Mn surrogates the targeted delivery and release of nanoencapsulated ATO. Schematic illustrations: (**a)** preparation of liposomal nanohybrids of arsenite-manganese functionalized with PS-targeting PGN635 F(ab’)_2_ (PS-L-AsMn) (**b**) PS-L-AsMn that can be seen as negative contrast on a T_2_-w image, binds specifically to PS-exposed tumor cells or tumor vascular endothelial cells (ECs) and becomes internalized into the cells. After exposure to a low pH in endosome-lysosome system, ionic As^3+^ and Mn^2+^ are released from the As-Mn complex, revealed as bright T_1_ contrast.

**Table 1:** Characteristics of arsenic liposomes functionalized with antibody fragments.

**Nanoprobe**	**Antibody modified (nmol)/lipid (mg)**	**Mean size (nm)**	**Zeta potential (mV)**	**Polydispersity index, PDI**
PS-L-AsMn	11.45 ± 0.65	92.44 ± 2.58	−2.84 ± 0.12	0.11 ± 0.02
Aur-L-AsMn	11.26 ± 1.17	93.72 ± 2.34	−2.82 ± 0.19	0.12 ± 0.02

Data were represented as the mean ± standard deviation. Each assay was repeated thrice.
