# Supplementary material for: Convertible MRI contrast: Sensing the delivery and release of anti-glioma nano-drugs
Source: Sci Rep. 2015 May 12;5:9874. doi: 10.1038/srep09874 (PMC4428068; doi:10.1038/srep09874)
Supplement: Supplementary Information [file srep09874-s1.pdf]

# Convertible MRI contrast: Sensing the delivery and release of anti-glioma nano-drugs

Liang Zhang[1], Zhongwei Zhang[1], Ralph P. Mason[1], Jann N. Sarkaria[2], Dawen Zhao[1*]

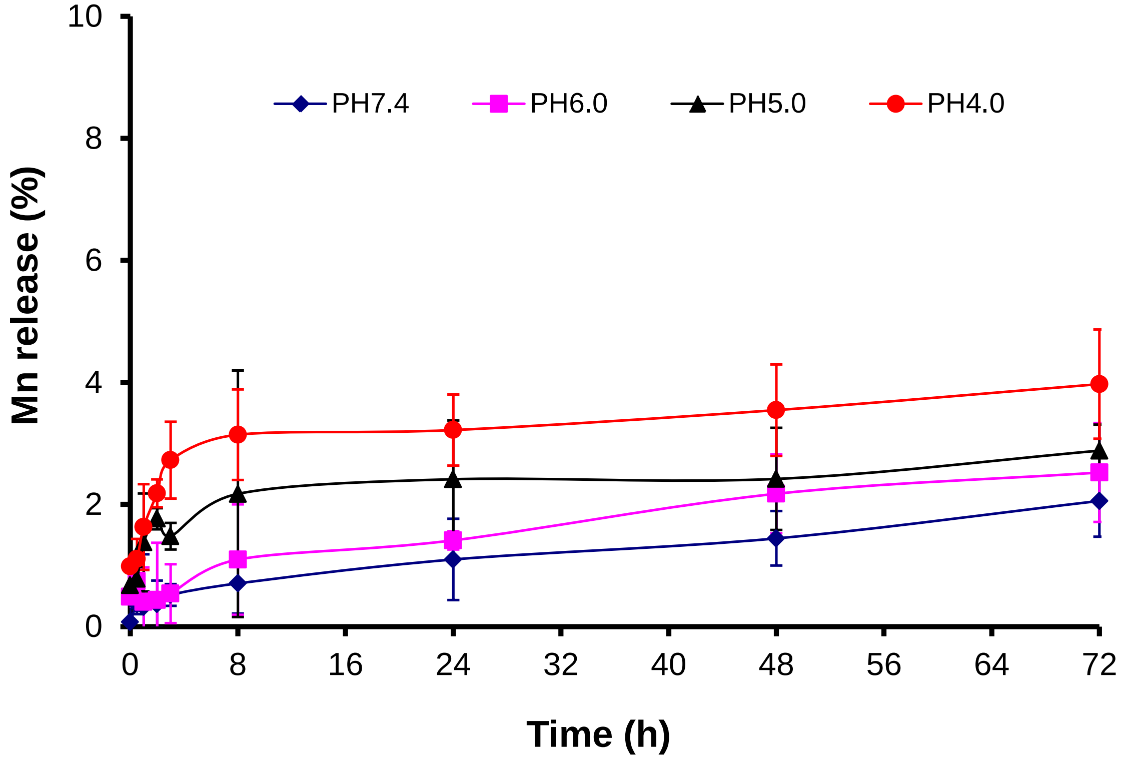


**Fig. S1 Release profiles of $Mn^{2+}$.** Time course of $Mn^{2+}$ release from liposomes at various pHs ranging from 7.4 to 4 was plotted. There was less than 5% leakage of $Mn^{2+}$ at 72h. Compared with the As ions (Fig. 2e), the transitional divalent metal ions ($Mn^{2+}$) have much limited membrane permeability.

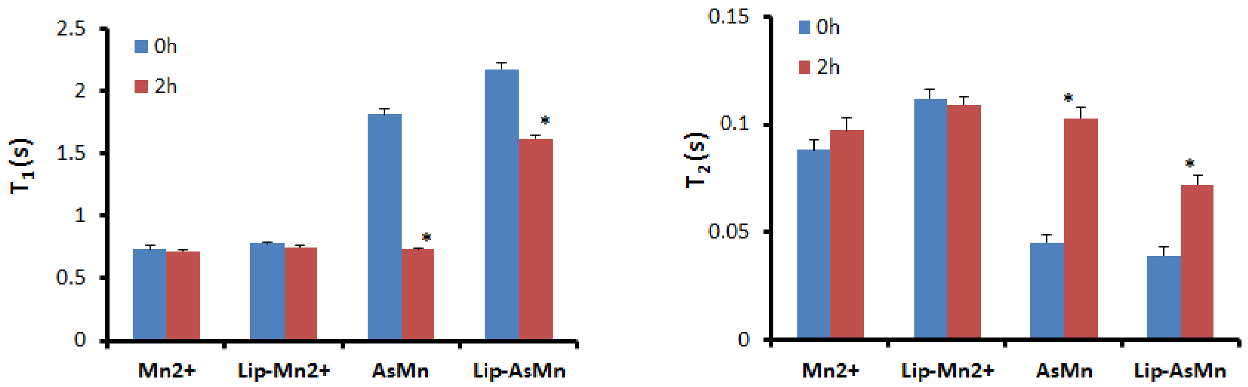


**Fig. S2 MRI contrast conversion in response to an acidic pH.** MRI measurements of $T_1$ and $T_2$ were conducted on the four Mn-containing materials $Mn^{2+}$, Lip-$Mn^{2+}$, AsMn and Lip-AsMn (0.1 mM Mn) before and 2h after exposure to pH5. **Left:** Two hours later, $T_1$ data revealed significantly shortened $T_1$ (T1-w SI↑) in AsMn and Lip-AsMn (*$p < 0.05$), while essentially no changes in $Mn^{2+}$ and Lip-$Mn^{2+}$. The magnitude of $T_1$ drop was smaller in Lip-AsMn than that of AsMn. Compared to $Mn^{2+}$, Lip-$Mn^{2+}$ has a longer $T_1$, implicating the restricted exchange between extraliposomal water and $Mn^{2+}$ in the core of liposome. **Right:** Significant increase on $T_2$ was detected in AsMn and Lip-AsMn (*$p < 0.05$), which may correlated with the reduced susceptibility effect due to dissociation of the AsMn complex. To be noted, liposomes maintained their integrity 2h after pH5.

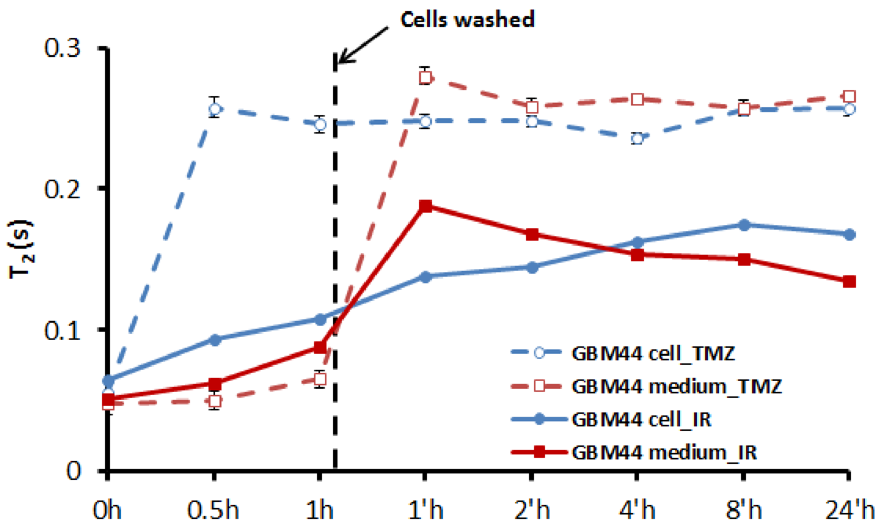


**Fig. S3 MRI of $T_2$ dynamics of GBM44 cells pre-treated with TMZ or IR.** $T_2$ changes corresponding to those GBM44 cells studied in Fig. 5d were quantified. In line with the previous histological and MRI studies, the uptake of PS-L-AsMn by TMZ-treated GBM44 cells was minimal, reflected by the immediately separation of $T_2$ curve of the cell compartment from the medium. After the removal of the unbound PS-L-AsMn at 1h, the two lines became closer. By contrast, GBM44 cells pre-treated with IR showed gradual increase on $T_2$, coinciding with the dynamic process of the binding and internalization of PS-L-AsMn and subsequent release of $Mn^{2+}$.

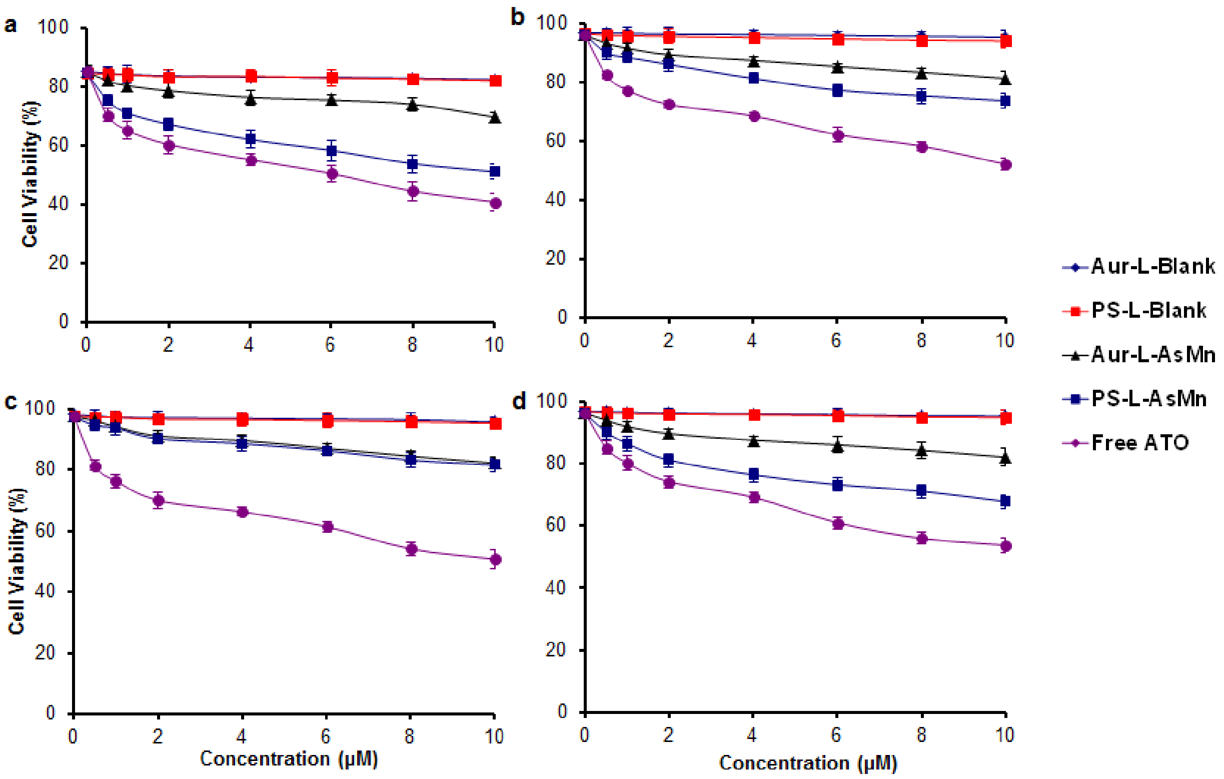


**Fig. S4 Cell toxicity of PS-L-AsMn.** GBM16 (**a** and **b**) and GBM44 (**c** and **d**) cells were pretreated with TMZ (50 µM, **a** and **c**) or irradiation (6 Gy, **b** and **d**) 24h before incubation with free ATO or various liposomal formulations for 4h. The cells were then washed and continued to culture in new medium. The cell survival assay at 72h revealed that the free ATO had the highest cell killing in each group. Prolonged treatment with PS-L-AsMn induced more dead cells, as compared to the 1h treatment in Fig. 6. However, there was also significantly increased cell death in the Aur-L-AsMn groups, indicating the non-specific uptake of liposomal ATO.
